# TopBP1 Interacts with BLM to Maintain Genome Stability but Is Dispensable for Preventing BLM Degradation

**DOI:** 10.1016/j.molcel.2015.02.012

**Published:** 2015-03-19

**Authors:** Andrew N. Blackford, Jadwiga Nieminuszczy, Rebekka A. Schwab, Yaron Galanty, Stephen P. Jackson, Wojciech Niedzwiedz

**Affiliations:** 1 The Weatherall Institute of Molecular Medicine, University of Oxford, Oxford OX3 9DS, UK; 2 The Gurdon Institute and Department of Biochemistry, University of Cambridge, Cambridge CB2 1QN, UK; 3 Institute of Biochemistry and Biophysics, PAS, 02-106 Warsaw, Poland; 4 The Wellcome Trust Sanger Institute, Hinxton, Cambridge CB10 1SA, UK

## Abstract

The Bloom syndrome helicase BLM and topoisomerase-IIβ-binding protein 1 (TopBP1) are key regulators of genome stability. It was recently proposed that BLM phosphorylation on Ser338 mediates its interaction with TopBP1, to protect BLM from ubiquitylation and degradation (Wang et al., 2013). Here, we show that the BLM-TopBP1 interaction does not involve Ser338 but instead requires BLM phosphorylation on Ser304. Furthermore, we establish that disrupting this interaction does not markedly affect BLM stability. However, BLM-TopBP1 binding is important for maintaining genome integrity, because in its absence cells display increased sister chromatid exchanges, replication origin firing and chromosomal aberrations. Therefore, the BLM-TopBP1 interaction maintains genome stability not by controlling BLM protein levels, but via another as-yet undetermined mechanism. Finally, we identify critical residues that mediate interactions between TopBP1 and MDC1, and between BLM and TOP3A/RMI1/RMI2. Taken together, our findings provide molecular insights into a key tumor suppressor and genome stability network.

## Highlights

• Contrary to the findings of Wang et al. (2013), TopBP1 does not maintain BLM levels
• BLM phosphorylation on Ser304, not Ser338, is required for TopBP1 binding
• Disrupting BLM-TopBP1 binding causes chromosome instability
• Crucial residues mediating TopBP1-MDC1 and BLM-TOP3A/RMI interactions identified

## Graphical Abstract

**Figure:**
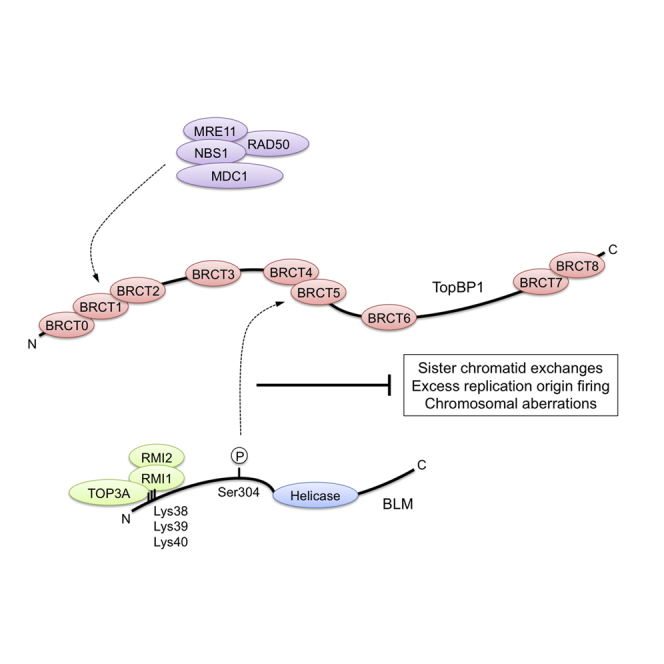

## Teaser

Wang et al. (2013) proposed that the BLM helicase and TopBP1 interact via phosphorylated Ser338 of BLM, and that TopBP1 shields BLM from degradation. In contrast, Blackford et al. reveal that the BLM-TopBP1 interaction involves Ser304 of BLM, not Ser338, and that TopBP1 has no role in stabilizing BLM.

## Introduction

TopBP1 is an essential protein with key roles in DNA replication and DNA damage responses (Wardlaw et al., 2014). It has no known enzymatic activity but contains nine BRCT domains and a C-terminal region that can stimulate the ATR checkpoint kinase (Kumagai et al., 2006). While most BRCT domains are phosphoprotein binding modules, some can interact in a phosphorylation-independent manner or recognize other molecules such as poly(ADP)ribose or DNA (Leung and Glover, 2011). TopBP1 has multiple binding partners for some of its BRCT domains, indicating that it exists in several discrete complexes. TopBP1-interacting proteins that have been reported to bind to specific BRCT domains include RAD9, Treslin, and NBS1 to TopBP1 BRCT1 (Delacroix et al., 2007; Kumagai et al., 2010; Lee et al., 2007; Yoo et al., 2009); 53BP1 and MDC1 to BRCT5 (Cescutti et al., 2010; Wang et al., 2011); and FANCJ (also known as BRIP1) to BRCT7 (Gong et al., 2010), although the mechanistic roles that these interactions play in TopBP1 functions are not yet clear.

Bloom syndrome is a rare autosomal recessive disorder caused by mutations in the gene encoding the BLM helicase, and is characterized by growth retardation, immunodeficiency, hypersensitivity to sunlight, and cancer predisposition (Bizard and Hickson, 2014). Cells from Bloom syndrome patients display multiple signatures of genome instability, including increased sister chromatid exchanges (SCEs) and chromosomal abnormalities (Chaganti et al., 1974; German et al., 1965). BLM performs its functions in a complex with topoisomerase IIIα (TOP3A), RMI1, and RMI2, which together form a “dissolvasome” complex capable of resolving homologous recombination (HR) intermediates to prevent genetic crossover events (Bizard and Hickson, 2014). Accordingly, BLM may act as a tumor suppressor primarily by preventing crossovers between homologous chromosomes that could lead to loss of heterozygosity. BLM also contributes to DNA-end resection to produce single-stranded DNA tracts for HR (Gravel et al., 2008), and BLM-deficient cells display DNA replication fork instability and excessive origin firing, indicating that BLM is an important regulator of replication dynamics (Davies et al., 2007; Rao et al., 2007).

We identified BLM as a TopBP1-interacting protein and found that BLM binding requires BRCT domain 5 of TopBP1, in agreement with a recent report (Wang et al., 2013). However, we demonstrate that phosphorylated Ser304 of BLM is in fact the target of this BRCT domain, rather than Ser338, as proposed by Wang et al. Also in contrast to that report, we find that neither TopBP1 loss nor disruption of the BLM-TopBP1 interaction has any discernible effect on BLM protein stability. Importantly, we establish that BLM-TopBP1 binding promotes genome stability, as disrupting the interaction in cells leads to increases in SCEs, replication origin firing, and chromosomal aberrations. Taken together, we conclude that although TopBP1 cooperates with BLM to maintain genome stability, it does so not by maintaining BLM protein levels, but via a different, as-yet-undefined mechanism.

## Results

### BLM Interacts with TopBP1 via BRCT5

**Figure 1:**
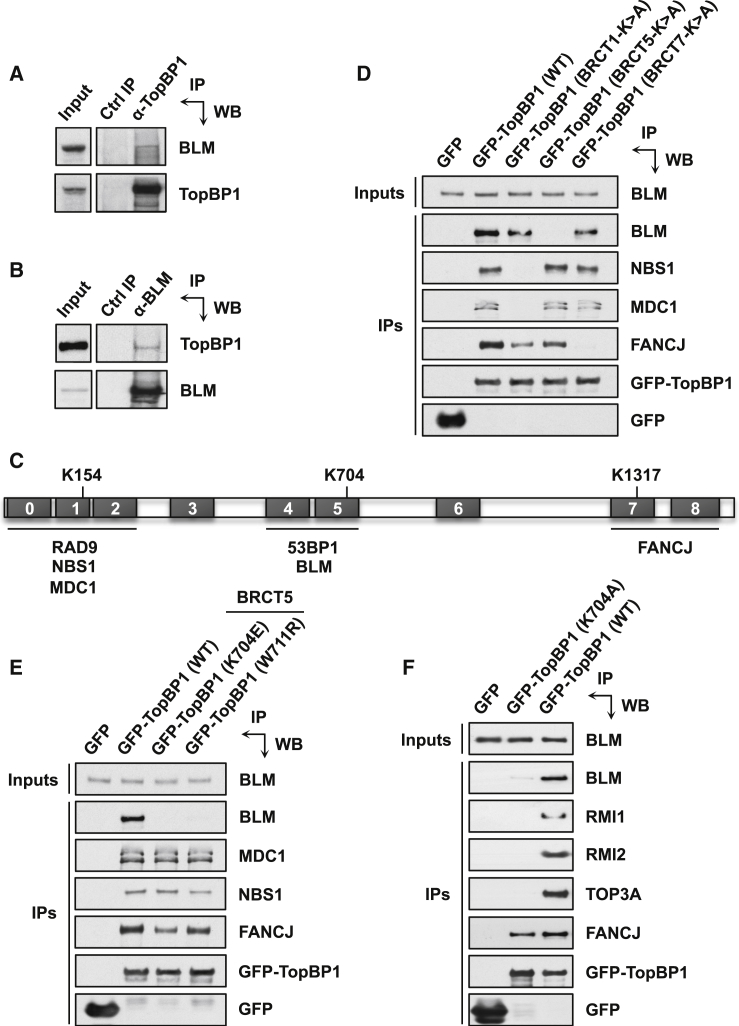
BLM Interacts with TopBP1 via BRCT5 (A) TopBP1 immunoprecipitates from 293FT cell extracts contain BLM. (B) BLM immunoprecipitates from 293FT cell extracts contain TopBP1. (C) Schematic showing TopBP1 BRCT domain layout. Black numbered boxes represent BRCT domains. K154, K704, and K1317 are the key phosphopeptide binding lysines in BRCT domains 1, 5, and 7, respectively. The names of known TopBP1-binding partners are shown below the BRCT domains they interact with. (D) Effect of point mutations in TopBP1 BRCT domains 1, 5, and 7 on its binding to NBS1, MDC1, FANCJ, and BLM compared to wild-type (WT). Pull-downs were carried out from 293FT cells transiently transfected with the indicated plasmids 24 hr later. (E) Effect of two different point mutations in TopBP1-BRCT5 on binding to MDC1 and BLM. NBS1 and FANCJ are positive controls as they bind to TopBP1 BRCT domains 1 and 7, respectively. (F) Mutation of TopBP1-BRCT5 abrogates binding to Bloom syndrome complex members. See also Figure S1.

We identified BLM as a candidate TopBP1 interactor by mass spectrometric analyses of TopBP1-associated proteins (see Figure S1 available online). To validate this interaction, we carried out coimmunoprecipitations from cell extracts and found that we could readily detect BLM by western blotting of TopBP1 immunoprecipitates (Figure 1A). Consistent with this, TopBP1 was detected in reciprocal BLM immunoprecipitates from cell extracts (Figure 1B), thus confirming that the two proteins likely exist in a complex together in cells.

To gain insight into the function of the BLM-TopBP1 interaction, we needed to map their reciprocal binding sites. Usually, one BRCT in a tandem unit contains a conserved lysine residue that directly contacts the phosphate group of a modified protein ligand (Leung and Glover, 2011). Accordingly, mutating such a lysine drastically reduces ligand-binding affinity. In TopBP1, these residues are Lys154 in BRCT1, Lys704 in BRCT5, and Lys1317 in BRCT7 (Cescutti et al., 2010). We therefore designed GFP-tagged TopBP1 constructs containing lysine-to-alanine mutations in BRCT domains 1, 5, and 7 (Figure 1C), as we considered that these domains were most likely to mediate the BLM interaction. To validate this approach, we probed for the presence of previously reported TopBP1-binding proteins whose interactions have been mapped to defined BRCT domains (Figure 1D). As expected, mutation of BRCT1 specifically prevented NBS1 binding (Yoo et al., 2009), and mutation of BRCT7 inhibited FANCJ binding (Gong et al., 2010). However, MDC1 binding was apparently unaffected by mutating TopBP1-BRCT5; note that we used a validated antibody that specifically recognizes MDC1 (Stewart et al., 2003). This was unexpected based on a previous report showing a requirement for this domain in mediating the TopBP1-MDC1 interaction (Wang et al., 2011). Furthermore, when we analyzed one of the same TopBP1-BRCT5 mutants used in that report (W711R), we found that it behaved identically to a Lys704 mutant, in that it did not affect MDC1 interaction (Figure 1E). Instead, MDC1 behaved as NBS1 in its binding profile, indicating that MDC1 in fact interacts with TopBP1 via BRCT1. This conclusion is consistent with the observations that most cellular MDC1 associates with the MRE11-RAD50-NBS1 (MRN) complex (Goldberg et al., 2003) and that TopBP1-BRCT1 is needed for binding to this complex, and with the suggestion that MDC1 could mediate TopBP1-MRN interactions (Yoo et al., 2009).

We next assessed whether any of our TopBP1 BRCT mutants were compromised in their ability to interact with BLM. Strikingly, only BRCT5 mutants lost the ability to bind BLM (Figures 1D and 1E), thus confirming that this BRCT domain mediates the TopBP1-BLM interaction, and suggesting that a phosphorylated residue in BLM or an associated protein might mediate this interaction. To explore whether TopBP1 is a component of the BLM dissolvasome, we examined binding of the other members of this complex (TOP3A, RMI1, and RMI2) to TopBP1. Like BLM, all these proteins interacted with wild-type TopBP1, but not with the BRCT5 mutant (Figure 1F), thus indicating that TopBP1 is a member of the dissolvasome complex.

### BLM Ser304 Phosphorylation Mediates a Direct Interaction with TopBP1

**Figure 2:**
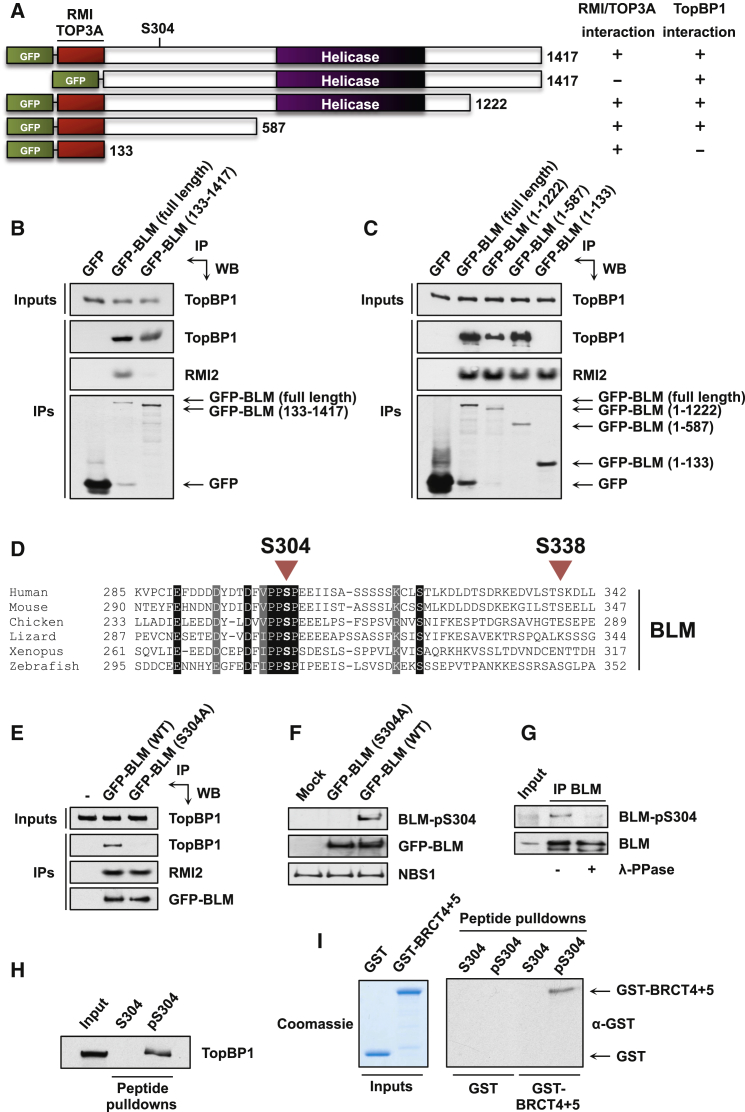
BLM Ser304 Phosphorylation Mediates Direct Binding to TopBP1-BRCT5 (A) Schematic of the GFP-tagged BLM constructs used in this study. (B) The N-terminal 132 residues of BLM are required for binding to TOP3A and RMI2 but not TopBP1. Pull-downs were carried out from 293FT cells transiently transfected with the indicated plasmids. (C) The binding site for TopBP1 is located within residues 133–587 of BLM. RMI2 is a positive control for binding to the N terminus of BLM. (D) Sequence alignment showing the evolutionary conservation of the BLM region containing Ser304 and Ser338. (E) Mutation of Ser304 specifically abrogates binding to TopBP1. Pull-downs were carried out from U2OS cells stably expressing the indicated proteins. (F) The BLM-pS304 antibody does not recognize BLM-S304A. 293FT cells were transiently transfected with the indicated plasmids. NBS1 is a loading control. (G) Ser304 is phosphorylated in vivo. BLM immunoprecipitates from U2OS cells were mock treated or treated with lambda phosphatase (λ-PPase). (H) BLM residues 297–311 are sufficient for interaction with TopBP1 when Ser304 is phosphorylated. Streptavidin beads were incubated with biotinylated peptides before addition to HeLa nuclear extracts for pull-downs. (I) TopBP1 BRCT domains 4 and 5 interact directly with BLM peptides phosphorylated on Ser304. Streptavidin beads were incubated with biotinylated peptides before mixing with GST-tagged BRCT domains 4 and 5 or GST alone. See also Figure S2.

To establish whether BLM itself or one of its binding partners was mediating BLM-TopBP1 binding, we took advantage of the fact that the N-terminal 132 residues of BLM are required for its interaction with the dissolvasome (Hu et al., 2001). Importantly, deleting this region led to the expected abrogation of TOP3A and RMI2 binding, but TopBP1 binding was unaffected (Figures 2A and 2B). This result indicated that TopBP1 can associate with BLM in the absence of other dissolvasome members, thereby strongly implicating BLM as the mediator of TopBP1 binding to this complex.

To determine the region of BLM that binds TopBP1, we examined binding of TopBP1 to a series of GFP-tagged BLM truncation mutants (Figure 2A). These studies showed that a BLM N-terminal region encompassing residues 133–587 was necessary for TopBP1 binding (Figure 2C). This region of BLM is not well conserved overall in vertebrates and may therefore be largely unstructured, but we noticed within it a potentially phosphorylatable motif centered on a highly conserved serine residue (Ser304 in human BLM; Figures 2D and S2). As BRCT domains can interact with phosphorylated proteins, we examined whether this residue was necessary for TopBP1 binding. Strikingly, mutating BLM Ser304 to alanine abolished TopBP1 binding but had no discernible effect on interaction between BLM and RMI2 (Figure 2E). These results thus established that Ser304 is specifically required for BLM to interact with TopBP1, and suggested that this residue might be phosphorylated. In accord with these findings and the predicted phospho-dependent nature of BLM-TopBP1 binding, when we used a phospho-specific antibody raised against a phosphopeptide encompassing BLM-Ser304 (Leng et al., 2006), it recognized wild-type BLM, but not the BLM S304A mutant (Figure 2F). Consistent with this site being phosphorylated in cells, the antibody detected endogenous BLM immunoprecipitated from cells in a manner that was inhibited by phosphatase treatment (Figure 2G).

To investigate whether the TopBP1-BLM interaction was direct, we used biotinylated nonphosphorylated peptides or Ser304-phosphorylated BLM peptides in interaction studies. Importantly, only the phosphopeptide retrieved TopBP1 from nuclear extracts (Figure 2H), indicating that this conserved motif in BLM is sufficient to mediate the TopBP1 interaction when phosphorylated on Ser304. In addition, recombinant GST-tagged TopBP1 protein comprising BRCT domains 4 and 5 bound to the phosphorylated Ser304 peptide, but not the nonphosphorylated peptide, whereas GST alone bound to neither (Figure 2I). Taken together with our other findings, these results established that the TopBP1-BLM interaction involves direct binding of phosphorylated BLM-Ser304 to TopBP1-BRCT5.

### Ser338 of BLM Is Not Required for TopBP1 Binding

**Figure 3:**
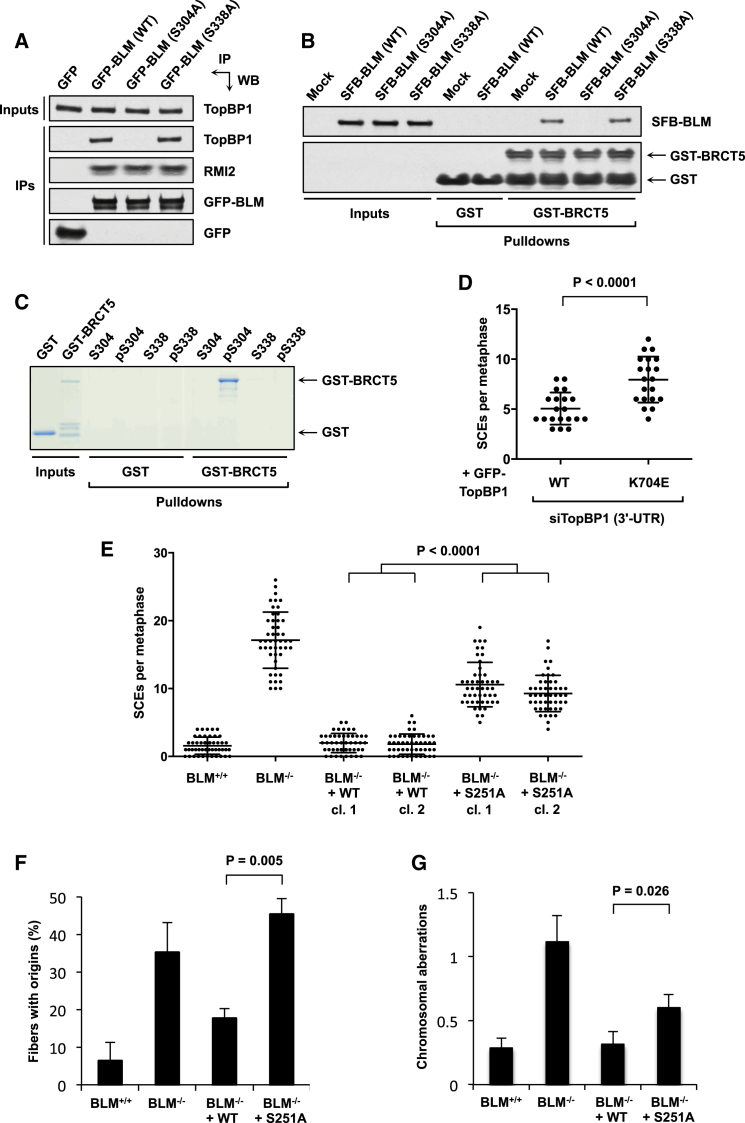
The BLM-TopBP1 Interaction Promotes Genome Stability and Requires Ser304 but Not Ser338 of BLM (A) Mutation of BLM Ser338 to alanine does not affect its interaction with endogenous TopBP1. Pull-downs were carried out from 293FT cells transiently transfected with the indicated plasmids. (B) Mutation of BLM Ser338 to alanine does not affect its interaction with recombinant GST-tagged TopBP1-BRCT5. Pull-downs were carried out using GST proteins bound to glutathione beads incubated with lysates from 293FT cells transiently transfected with the indicated plasmids. (C) TopBP1-BRCT5 interacts directly with BLM peptides encompassing phosphorylated Ser304 but not Ser338. Streptavidin beads were incubated with biotinylated peptides before mixing with GST-tagged BRCT5 or GST alone. (D) Analysis of SCEs in U2OS cells depleted of endogenous TopBP1 with siRNAs targeting the 3′ UTR and expressing wild-type or K704E TopBP1. A minimum of 20 metaphases was scored per experiment. Significance was determined using the Mann-Whitney *U* test. (E) Analysis of SCEs in DT40 cells. A minimum of 50 metaphases was scored per experiment. Significance was determined using the Mann-Whitney *U* test. “cl.,” clone. (F) DNA fiber analyses to measure origin firing. DT40 cells were treated with 2.5 μM camptothecin for 90 min in the presence of IdU, washed, and then released into drug-free medium containing CldU for 15 min. A minimum of 200 fibers were scored per experiment. Mean values of three independent experiments are shown ± SEM. Significance was determined using Student’s two-tailed t test. (G) Analysis of chromosomal aberrations. Mitotic spreads were prepared from DT40 cells treated with 2 μM aphidicolin for 12 hr. A minimum of 35 metaphases was scored per experiment. Significance was determined using Mann-Whitney *U* test. See also Figure S3.

Our conclusion that Ser304 is the critical residue of BLM that interacts with TopBP1 BRCT domain 5 differed from that of a recent report describing a role for Ser338 in this binding (Wang et al., 2013). We initially considered that our respective studies were not necessarily in disagreement, since it might have been that phosphorylation of multiple residues was required for the BLM-TopBP1 interaction. We therefore tested whether we could replicate a role for BLM-Ser338 in mediating TopBP1 binding. To do this, we expressed GFP-tagged wild-type BLM or derivatives in which Ser304 or Ser338 was mutated to alanine (S304A and S338A, respectively) in cells and assessed their abilities to retrieve endogenous TopBP1 from cell extracts (Figure 3A). In contrast to BLM-S304A, the S338A mutant behaved as wild-type in its ability to bind TopBP1, indicating that Ser338 is not required for the BLM-TopBP1 interaction. Analyses using cells synchronized in S phase produced similar results (Figure S3A).

We considered the possibility that the contradictions between our results and those of Wang et al. might be due to the different methodologies used. We therefore incubated recombinant GST-tagged TopBP1-BRCT5 purified from bacteria with lysates from human cells transfected with plasmids expressing S/Flag/streptavidin-binding peptide (SFB) triple-tagged BLM as described in the previous study (Wang et al., 2013). Notably, the wild-type SFB-BLM and its S304A and S338A mutant derivatives behaved in the same manner as the respective GFP-tagged proteins with regard to TopBP1 binding, in that wild-type and S338A BLM bound to GST-BRCT5 in similar amounts, whereas the S304A mutant was severely compromised in this regard (Figure 3B).

Next, we examined the relative abilities of peptides encompassing phosphorylated or nonphosphorylated Ser304 or Ser338 of BLM to interact with GST-BRCT5. While the phospho-S304 peptide readily bound the TopBP1 fusion protein, neither the phospho-S338 peptide nor the nonphosphorylated peptides were able to interact with it detectably (Figure 3C). Taken together, our findings thus established that BLM Ser304, not Ser338, binds to BRCT domain 5 of TopBP1.

### The BLM-TopBP1 Interaction Promotes Genome Stability

BLM-deficient cells display characteristically high levels of SCEs (Chaganti et al., 1974). To address the potential biological function of BLM-TopBP1 binding, we tested whether the interaction was required to suppress SCEs. First, we examined the phenotypes of U2OS cells stably expressing wild-type or the K704E BRCT5 mutant TopBP1 and treated with an siRNA targeting the TopBP1 3′ untranslated region (UTR) to deplete the endogenous protein (Figure S3B). Notably, cells expressing TopBP1-K704E displayed significantly elevated SCEs compared to cells expressing wild-type TopBP1 (Figure 3D). Next, we employed *BLM*^−/−^ chicken DT40 cells as a complementation system by stably transfecting these with vectors encoding wild-type BLM or BLM-S251A (equivalent to S304A in human BLM; Figure 2D). We confirmed that the BLM-S251A mutation abrogated TopBP1 binding (Figure S3C), thus revealing that the BLM-TopBP1 interaction is highly conserved in vertebrates. As expected, *BLM*^−/−^ cells displayed significantly higher levels of SCEs than *BLM*^+/+^ cells. Importantly, this phenotype was fully reversed by reintroducing wild-type BLM but not the S251A mutant in multiple clones (Figure 3E). These findings therefore established that the BLM-TopBP1 interaction contributes to suppression of SCEs in vertebrates.

Replication origins in BLM-deficient cells fire more frequently than in wild-type cells, probably as a consequence of problems in replication fork dynamics (Davies et al., 2007; Rao et al., 2007). We therefore examined whether cells in which BLM-TopBP1 binding is disrupted were similarly defective in this process by using DNA fiber analyses. Compared to cells expressing wild-type BLM, we detected significantly higher origin firing in *BLM*^−/−^ cells as well as in cells expressing BLM-S251A (Figure 3F). These data thus further supported the notion that interaction with TopBP1 is required for BLM to function properly.

As BLM-deficient cells display increased chromosomal aberrations (German et al., 1965), we investigated whether this was also the case in cells in which BLM-TopBP1 binding was disrupted. This revealed that there was a significant increase in the frequency of such aberrations in *BLM*^−/−^ cells after aphidicolin treatment, which was corrected by expression of wild-type BLM but only partially with BLM-S251A (Figure 3G). Taken together, these data confirmed that the BLM-TopBP1 interaction is important for maintenance of genome stability.

### TopBP1 Does Not Protect BLM from Degradation

**Figure 4:**
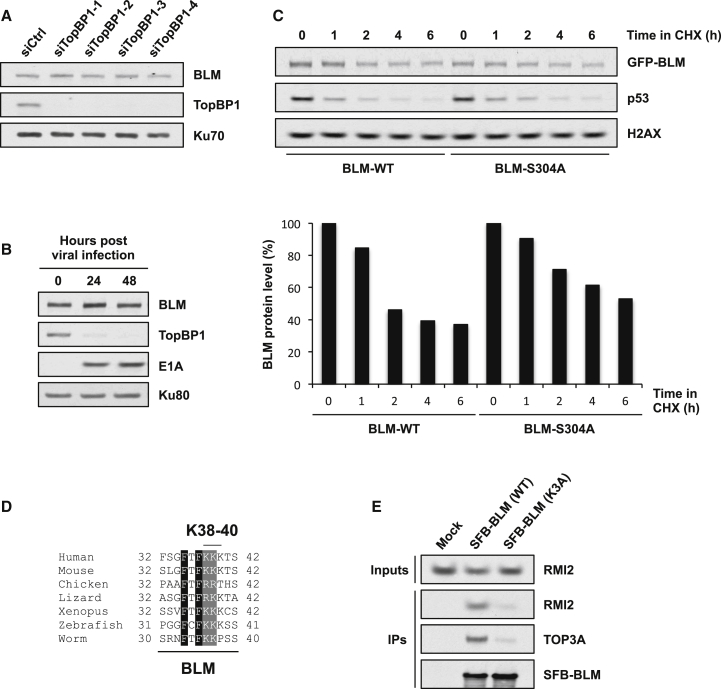
TopBP1 Does Not Protect BLM from Degradation (A) TopBP1 depletion does not affect BLM levels. HeLa cells were transfected with the indicated siRNAs and harvested for western blotting 3 days later. (B) Adenovirus-induced proteasomal degradation of TopBP1 does not affect BLM levels. U2OS cells were infected with the *hr*703 adenovirus and harvested at the indicated times for western blotting. E1A is a control for adenovirus infection. (C) BLM-TopBP1 interaction does not maintain BLM stability. Cycloheximide (CHX) was added to U2OS cells stably expressing GFP-BLM proteins for the indicated times before harvesting for western blotting. The graph shows the level of BLM at the time points indicated as a percentage of untreated (0 h). Quantification was performed using ImageJ. (D) Sequence alignment showing the evolutionary conservation of the BLM region containing Lys38, Lys39, and Lys40. (E) Mutation of BLM Lys38, Lys39, and Lys40 to alanine (K3A) disrupts binding to TOP3A and RMI2. 293FT cells were transfected with the indicated plasmids and harvested for pull-downs 24 hr later. See also Figure S4.

It was suggested that BLM interaction with TopBP1 is required to prevent ubiquitylation and subsequent proteasomal degradation of BLM (Wang et al., 2013). We therefore assessed whether we could also observe BLM destabilization in absence of TopBP1. To do this, we first verified that our BLM antibody was specific by showing that it recognized a protein species that is absent in Bloom syndrome cells and in cells treated with an siRNA targeting the BLM mRNA (Figures S4A and S4B). We then transfected cells with TopBP1 siRNAs with sequences that were identical to those used by Wang et al., as well as two additional ones of our own design. Strikingly, all four siRNAs depleted TopBP1 to near-undetectable levels in HeLa cells but had no appreciable effect on BLM protein stability (Figure 4A). Similar results were obtained in U2OS cells (data not shown), suggesting that TopBP1 plays no discernible role in regulating BLM levels. To seek to confirm this, we used an alternative method to reduce TopBP1 expression in cells. Some human adenoviruses promote TopBP1 degradation in infected cells by hijacking a Cullin-based E3 ubiquitin ligase with the viral E4orf6 protein and directing it toward TopBP1 (Blackford et al., 2010). We therefore used one such virus, *hr*703, to examine BLM levels during infection. Although TopBP1 was rapidly and almost completely degraded in cells infected with *hr*703, BLM levels actually increased slightly (Figure 4B). Finally, we compared the half-lives of wild-type and S304A BLM in cells treated with the translation inhibitor, cycloheximide. Ensuing results demonstrated that the half-lives of both proteins were roughly equivalent, with the S304A mutant in fact being slightly more stable (Figure 4C). Synchronization of cells in S phase yielded similar results (Figure S4C). Based on these experiments, we concluded that TopBP1 plays no significant role in controlling BLM protein levels.

Wang et al. suggested that BLM degradation might be necessary in G1 cells to prevent DNA-end resection and initiation of HR in the absence of a sister chromatid. This was based on their observations that cells stably overexpressing mutant nondegradable BLM with three N-terminal lysines (Lys38, Lys39, and Lys40) mutated to alanine (“K3A”) were hypersensitive to ionizing radiation, showed increased phosphorylation of CHK1 and RPA, and had a reduced frequency of random plasmid integration (Wang et al., 2013). Notably, lysines 38, 39, and 40 are conserved in most vertebrate BLM proteins (Figure 4D) and lie within the region required for BLM association with TOP3A and RMI1/2 (Figures 2A–2C). We therefore considered the possibility that the BLM K3A mutant was compromised in its binding to the dissolvasome. To test this, we compared binding of TOP3A and RMI2 to wild-type and K3A BLM (Figure 4E). We first noted that the K3A mutant was expressed at similar levels to wild-type BLM, suggesting that lysines 38, 39, and 40 do not control BLM turnover. Moreover, we found that substantially less TOP3A and RMI2 was associated with the K3A mutant compared to wild-type BLM. Therefore, we concluded that it is not possible to determine whether phenotypes seen in cells expressing BLM-K3A are due to the inability of this protein to associate with the dissolvasome, or because it cannot be ubiquitylated.

## Discussion

In this study, we identified and characterized the interaction between the Bloom syndrome helicase BLM and TopBP1, and investigated the consequences for cells when binding is disrupted. We found that BLM interacts directly with BRCT domain 5 of TopBP1 via a phosphorylated N-terminal residue (Ser304 in human BLM). This finding was surprising, given a recent report suggesting that Ser338 was important for TopBP1 binding (Wang et al., 2013). However, we note that Ser338 and its surrounding amino acid residues are not well conserved even in most mammals, but Ser304 and surrounding residues have been highly conserved throughout vertebrate evolution (Figure 2D). Accordingly, using similar assays and identical reagents in many cases to those employed by Wang et al., we clearly established that Ser338 does not play a major role in BLM-TopBP1 binding. Therefore, we conclude that it is phosphorylated Ser304, not Ser338, that interacts directly with TopBP1-BRCT5 in cells.

Significantly, while Wang et al. showed that TopBP1-BRCT5 mutations lead to increased SCEs as we did, they did not show this phenotype for cells expressing BLM-S338A. By contrast, we clearly observed increased SCEs in multiple *BLM*^−/−^ clones stably expressing BLM-S251A (the chicken equivalent of S304A). We also tested whether we could recapitulate another claim by Wang et al., that TopBP1 stabilizes BLM, but we were unable to do so. In our hands, BLM stability was normal in absence of TopBP1 using various methods, including when we used identical siRNA sequences to deplete TopBP1 in the same cell lines used by Wang et al. In addition, mutant BLM that cannot interact with TopBP1 was not less stable in our studies. Taken together, these data strongly suggest that TopBP1 binding does not protect BLM from proteasomal degradation, and that the BLM-TopBP1 interaction therefore maintains genome stability via another mechanism.

Our data cannot rule out an indirect role for TopBP1 in maintaining BLM stability, because they leave open the formal possibility that our TopBP1 siRNA depletions were not as efficient as those of Wang et al. Crucially, however, under experimental conditions where we observed increased SCEs in TopBP1-depleted cells, there was no effect on endogenous BLM protein stability. Thus, even if TopBP1 has some indirect role in maintaining BLM stability that we could not detect due to incomplete TopBP1 depletion (a possibility we consider unlikely), this would still not explain the chromosomal instability phenotypes we see in TopBP1-depleted cells or cells expressing BLM or TopBP1 binding mutants.

It was reported that ubiquitylation of lysines 38, 39, and/or 40 in BLM leads to its degradation, that this is prevented by TopBP1 binding, and that cells expressing BLM with these sites mutated (“K3A”) show signs of increased DNA-end resection in G1 (Wang et al., 2013). These three residues are quite well conserved in vertebrate BLM, but in some organisms they are replaced by arginine, suggesting that it is the positive charge on these residues that is functionally important rather than their ability to be ubiquitylated. Accordingly, we found that lysines 38, 39, and 40 of BLM do not obviously affect its protein levels. Instead, we found that the K3A mutant protein was defective in its association with dissolvasome components, which are required for BLM to promote DNA-end resection (Daley et al., 2014). We thus conclude that any defects observed in cells expressing the K3A BLM mutant could be due to lack of binding to the dissolvasome rather than its inability to be ubiquitylated.

A number of issues arise from our work that will be worthwhile to address in future studies. In particular, it remains to be determined which kinase phosphorylates BLM on Ser304 to promote its interaction with TopBP1, and how it is regulated. Finally, given that cells expressing mutant BLM that cannot bind TopBP1 display increased SCEs and chromosomal aberrations, our data may have important implications for understanding the increased cancer susceptibility and pathologies observed in Bloom syndrome patients.

## Experimental Procedures

### Cells and Chemicals

293FT, HeLa, U2OS, and DT40 cells were grown as described previously (Blackford et al., 2012). Wild-type (JB1) and Bloom syndrome (GM03403) lymphoblastoid cell lines (LCLs) were grown in RPMI-1640 (Sigma-Aldrich) supplemented with 10% fetal calf serum and 1× penicillin-streptomycin-glutamine (Life Technologies). Stable U2OS lines were established by selection in medium containing 0.5 mg/ml G418 (Life Technologies). All chemicals were from Sigma-Aldrich except lambda phosphatase (New England Biolabs).

### Antibodies, SDS-PAGE, and Western Blotting

See the Supplemental Information for antibodies used in this study. SDS-PAGE and western blotting were performed as described previously (Polo et al., 2012).

### Plasmids, siRNAs and Transfections

See the Supplemental Information for plasmids and siRNAs used in this study. Plasmids were transfected into DT40 by electroporation and human cells using Lipofectamine 2000 (Life Technologies) according to the manufacturer’s instructions. Small interfering RNAs (siRNAs) were transfected using Lipofectamine RNAiMAX (Life Technologies) according to the manufacturer’s instructions.

### Adenovirus Infections

Adenovirus infections were carried out as described (Blackford et al., 2008). The *hr*703 *E1B* mutant adenovirus was used rather than wild-type virus, because one of the *E1B* gene products induces BLM degradation (Orazio et al., 2011).

### Immunoprecipitations

For preparation of lysates for immunoprecipitations (IPs), cells were washed twice in phosphate-buffered saline (PBS) and lysed in IP buffer (100 mM NaCl, 0.2% Igepal CA-630, 1 mM MgCl_2_, 10% glycerol, 5 mM NaF, 50 mM Tris-HCl [pH 7.5]), supplemented with Complete EDTA-free protease inhibitor cocktail (Roche) and 25 U/ml Benzonase (Novagen). After nuclease digestion, NaCl and EDTA concentrations were adjusted to 200 mM and 2 mM, respectively, and lysates were cleared by centrifugation. Where appropriate, antibodies were added to a final concentration of 1 μg/mg lysate and incubated for 2 hr at 4°C. Lysates were then incubated with 10 μl of either GFP-Trap agarose beads (ChromoTek), anti-Flag M2 affinity gel (Sigma-Aldrich), or protein G Sepharose (GE Healthcare) for 2 hr with end-to-end mixing at 4°C. Immunoglobulin-antigen complexes were washed extensively before elution in 2× SDS sample buffer for SDS-PAGE.

### Recombinant Protein Purification and Peptide Pull-downs

Glutathione *S*-transferase (GST) proteins were purified as described (Blackford et al., 2008). Biotinylated peptides (Genosphere Biotechnologies) were bound to streptavidin-coupled Dynabeads M-280 (Life Technologies) before incubation with HeLa nuclear extracts (CilBiotech) or purified GST proteins for 2 hr with end-to-end mixing at 4°C. Beads were washed with peptide pull-down buffer (175 mM NaCl, 0.2% Igepal CA-630, 10% glycerol, 5 mM NaF, 1 mM EDTA, 50 mM Tris-HCl [pH 8.0], and protease inhibitors) before resuspension in 2× SDS sample buffer for SDS-PAGE. See the Supplemental Information for peptide sequences.

### GST Pull-downs

Pull-downs were performed as described (Wang et al., 2013).

### Analyses of DNA Fibers, SCEs, and Chromosomal Aberrations

Cells were prepared for analyses of DNA fibers, SCEs, and chromosomal aberrations as described (Niedzwiedz et al., 2004; Schwab et al., 2010).

## Author Contributions

A.N.B. carried out the majority of experimental work, with substantial contributions by J.N. and W.N.; R.A.S. performed DNA fiber experiments, Y.G. contributed stable cell lines, and the paper was written by A.N.B. with editing by S.P.J. and W.N.

## Supplemental Information

Document S1. Figures S1–S4 and Supplemental Experimental Procedures

Document S2. Article plus Supplemental Information
