# Supplementary material for: TopBP1 Interacts with BLM to Maintain Genome Stability but Is Dispensable for Preventing BLM Degradation
Source: Mol Cell. 2015 Mar 19;57(6):1133–41. doi: 10.1016/j.molcel.2015.02.012 (PMC4374139; doi:10.1016/j.molcel.2015.02.012)
Supplement: Document S1. Figures S1–S4 and Supplemental Experimental Procedures [file mmc1.pdf]

Molecular Cell, Volume *57*

Supplemental Information

TopBP1 Interacts with BLM to Maintain Genome Stability but Is Dispensable for Preventing BLM Degradation

Andrew N. Blackford, Jadwiga Nieminuszczy, Rebekka A. Schwab, Yaron Galanty, Stephen P. Jackson, and Wojciech Niedzwiedz

PROTEIN	PEPTIDES
TOPBP1	124
BLM	51
MDC1	49
RFC1	45
53BP1	25
RFC3	22
IWS1	22
TCOF1	21
RFC4	20

Figure S1 (related to Figure 1): Identification of TopBP1-associated proteins by mass spectrometry. Pulldowns were carried out from 293FT cells transiently transfected with plasmids expressing GFP-tagged TopBP1 or GFP as a control. The top hits found exclusively in the GFP-TopBP1 sample are shown in the table.

```
Human       133 DTALKKLEFSSSPDSLSTI-NDWDDMDDFDTSETSKSFVTPPQSHFVRVSTAQKSKKGKRNFFKAQLYTT
Mouse       133 VAVFKKLEFSSSA---DSL-SDWADMDDFDMSASDAF-ASLAKNPATRVSTAQKMKKTKRNFFKPPPRKA
Chicken      97 GSVSVSLPSSG--PAAYTG-DEWDDMDDFDLSGFEKKFS-RPAVLSP----------------KGPRT--
Lizard      125 QLENPKADHNSSYSSIINIDDVWDDIDDFEIARQEKKCSKSPASLSS---NTLLAKKSKAPKNQKPLTSE
Xenopus     122 DAASKKTGINTSFGSVTSL-EEWDDLDDFDTSVSPPKSH-AGKGG------------------KTPQK-C
Zebrafish   138 NDQTVELKHDI---SQLSF-NEWDDLDDFETPVKSRVAS-PVAGTST----------------KKPSVSD

Human           NTV----------KTDLPPPSSESEQIDLTEE---------------QKDDS-EWLSSDVICIDDGPIAE
Mouse           NAV----------KTDLTPPSPECLQVDLTKESEEEEEE------EEEAEGA-DCLSRDVICIDNDSASE
Chicken         ------------------------PQGRGLRVSKARADVGPQEHGSE-VSCSQGSSQGSLICLGDAGAPA
Lizard          QLVPLVSRQGSQL----CNEGFVNNKSNTIQIPDDDDDQ----------EEN-APGSQSVICLGPITS-H
Xenopus         K---NTSPVASFKIQSISPEGPTTEKHDCAKLLYDNNEVASEPRKNLHAKTA-ESPDQSLVCLASVEP-T
Zebrafish       Q---NTSSSCSSK----CEETKVNEESQA--------------------TET-ITAPKDVLSAANGVS-T

Human           VHINEDAQESDSL-KTH---------------------------LEDERD----NSEKKKNLEEAELHST
Mouse           ELTEKDTQESQSL-KAH---------------------------LGAERG----DSEKKSHEDEAVFHSV
Chicken         AAVLSDNGMEEARAATN---------------------------GGSGESQKLSNGEKSSQLEPGDV-GN
Lizard          DGSYEDVPANRPL-GSP---------------------------PGSKKVESLEI-RNDKGEKPGSA-CN
Xenopus         NLERDMCRNTDYL-G----------------------------------TDDLE-HDQETL---------
Zebrafish       ETAEREPEDS-PIKKSKRPKKSVQHTALLSDTEDEEIIHCVSPDTNQKNFKSVAVAEKDKWGEPNVI-D-

Human           EKVPCIE-FDDDDYDTDFVPPSPEEIISA-SSSSSKCLSTLKD--------LDTSDRK--EDVLST-SKD
Mouse           QNTEYFE-HNDNDYDIDFVPPSPEEIIST-ASSSLKCSSMLKD--------LDDSDKE--KGILST-SEE
Chicken         ELLADIE-LEED-DYLDVVPPSPEEELPS-FSPSVRNVSNIFK--------ESPTDGR--SAVHGTESEP
Lizard          DPEVCNE-SETE-DYVDFIPPSPEEEEAPSSASSFKSISYIFK--------ESAVEKTRSPQALK--SSS
Xenopus         -SQVLIE-EEDD-CEPDFIPPSPSDESLS-SPPVLKVISAQRKHKVSSLTDVNDCENT--TDH-------
Zebrafish       -SDDCEENNHYE-GFEDFIPPSPIPEEIS-LSVSDKEKSSSEP--------VTPANKK--ESS-------

Human           LLSKPEKMSMQEL----NPETSTDCDARQIS-LQQQLIHVMEHICKLIDTIPDDKLKLLDCGNELLQQRN
Mouse           LLSKPEEMTTHKS----DAGTSKDCDAQQIR-IQQQLIHVMEHICKLVDTVPTDELEALNCGTELLQQRN
Chicken         ELMAPKQPAAEQD-------SSAEHADKGLH-LEQQLYSVMEDICKLVDAIPLHELTSISCAKELLQQRE
Lizard          GASSGSRKAQEDSDEGGFSEVQCYLSGKPFP-SGQSLYSVMEDICKLVDSIPERDLRFLSCGSELIRHRD
Xenopus         LQ--GQS-------------VSTSLDS---K-VPSQLLTLMLEICDLVDKIPISELHVLSCGLDLKKKRD
Zebrafish       RSASGLP-------------APLDQSAKGLKGADDALFSVMESICCLVDTIPEHELIALTCGTELLLQRA

Human           IRRKLLTEVDF-NKSDASLLG--------SLWRYRPDSLDGPMEGDSCPTG---------------NSMK
Mouse           IRRKLLAEAGF-NGNDVRLLG--------SLWRHRPDSLDNTVQGDSCPVG---------------HPNK
Chicken         LRRKLLADSGA-LNTN---------------------SVNGPRNWKACVQQDPSSRPGTPLCSGPGRGVS
Lizard          YRYDGSLDSGVSFRLEE-------------------ENVDSPPGGMSTSTQ---------------PSVR
Xenopus         MRKRLLSNDSV-FRSSPADSSTVSLTSCTSSTQNRDFNVNAPKGAESLSGS---------------SVSK
Zebrafish       HRKRILAKGGS-SRTSH-------------------SDSVSTPG------------------------FLN

Human           ELNFSHLP---------SNSVSPGDCLLTTT-----LGKTGFSATR-----------KNLF---------
Mouse           ELNSPYLL---------SHSPSTEECLPTTT-----PGKTGFSATP-----------KNLF---------
Chicken         SVG----------STPK---STNLPSVLSRT-----VNSSSFSTIR-----------NQTLDKLDTSYSS
Lizard          KFG----------VFERHHNFHSTPSVVPAV-----QGTSNVSLSRFPG--------NSAL---------
Xenopus         VFKFNKLAVHDIGTKESENSANSAPNFMEKI-----GNKTSFSFRA-GG--------DSIM---------
Zebrafish       RPTFGVTP---------SNLT----SLTPVTSGKREGVKTGFSFRK-SIASVMSVGDESVF---------

Human           ----ER----------------------------PLFNTHLQKSFVSSNWAE--TP--------------
Mouse           ----ER----------------------------PLLNSHLQKSFVSSNWAE--TP--------------
Chicken         KETDQEVICL-EPAALPSPKVNGKGSTSLSRPSEASF---------NGSWCE--KP--------------
Lizard          ----EN-----------------------------GI-----------SPL-------------------
Xenopus         ----EN-----------------------------SFNF-------HSSVL-------------------
Zebrafish       ----EDSDCIINGVETPGGTWNPNSSTKISAG-RDTF---------NGSIQTLSKPESKTDKCYSRLSFN

Human           -RLGKKNESSYFPGN--VLTSTAV-----KDQNK-HTASIND-----LERE---TQPSYDIDNFDIDDFD
Mouse           -RMENRNESTDFPGS--VLTSTTV-----KAQSK-QAASGWN-----VERH---GQASYDIDNFNIDDFD
Chicken         -T-GRDSGNWRVPER--PTASTAL-----KAQ---HTAPAGNPASGCWD-V---NDTDFDLDHFDIDDFD
Lizard          -----QNASLSFPKR--GSSGERMWSENFMNPEMNVTAESDS-----LA-N---RAGNFDDDNFDIDDLN
Xenopus         -----SNSRFNTPQNEKPISSSTC-----TRP---YSQPIDD-----MD-N---PDLDFDIDNFDIEDLD
Zebrafish       ES-SNQTDLFYSPKR--VDSGSRNAD---SSVEI-NIAGSSS-----LR-TGAEPVDDFLLDDFDIDDFD

Human           DDD---DWEDIMHNLAA-SKSSTAAYQPI 587
Mouse           DDDDDDDWENIMHNFPA-SKSSTATYPPI 595
Chicken         EGW-----EEAVAPEAAPEAPPAPQWQPL 561
Lizard          ELD------EIIQLSGA-EQPLQASCLPA 569
Xenopus         DIH---CLDSPAAPSV--SSKNVPQYPTI 552
Zebrafish       ENDIPDYYEEPPSVLES-RNNSGVKTPSV 616
```

Figure S2 (related to Figure 2): Sequence alignment of BLM 133-587. Alignments were carried out using T-Coffee (http://tcoffee.crg.cat/apps/tcoffee/do:regular) and Boxshade (http://www.ch.embnet.org/software/BOX_form.html) programs. Conserved residues are highlighted in black, similar residues are highlighted in grey. Human S304 and equivalent residues in other organisms are shown in bold and indicated with a red triangle.

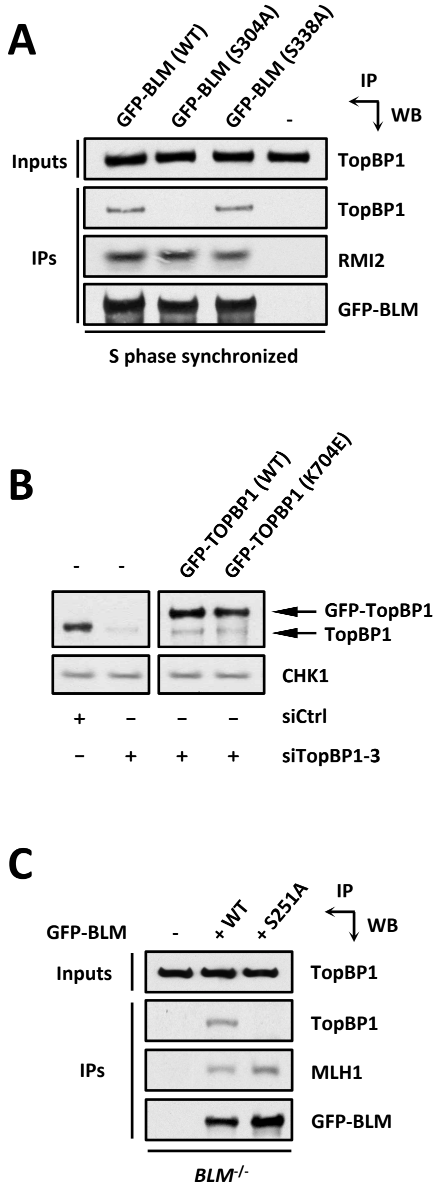

Figure S3 (related to Figure 3): Ser338 of BLM is not required for TopBP1 binding in S phase and generation of cells stably expressing point mutations of BLM and TopBP1. (**A**) Mutation of BLM Ser338 to alanine does not affect its interaction with endogenous TopBP1 in S phase. Pulldowns were carried out from 293FT cells transiently transfected with the indicated plasmids and released from a thymidine block. (**B**) Complementation of TopBP1-depleted cells with WT and K704E TopBP1. U2OS cells stably expressing GFP-tagged WT or K704E TopBP1 and their parental line were transfected with siRNA targeting firefly luciferase (siCtrl) or the TopBP1 UTR (siTopBP1-3). CHK1 is a loading control. (**C**) Ser251 is the chicken equivalent of Ser304 in human BLM. *BLM*$^{-/-}$ DT40 cells were stably complemented with the indicated plasmids. MLH1 was used as a positive control for BLM binding as it binds the C-terminus of BLM.

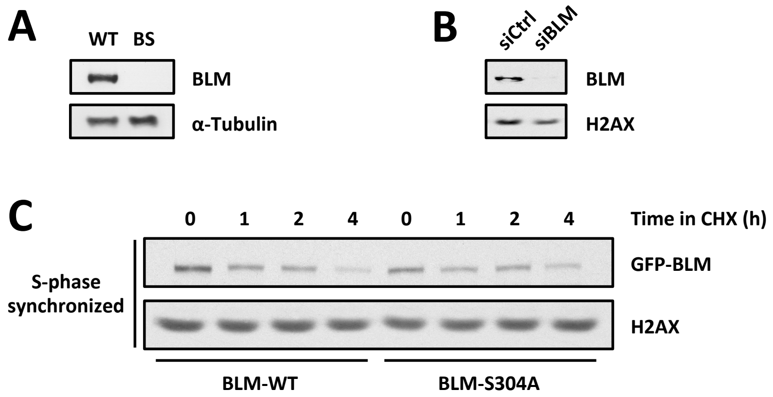

Figure S4 (related to Figure 4): Specificity of the BLM antibody used in this study and S phase stability of mutant BLM that cannot bind TopBP1. (**A**) Western blot of cell extracts from a Bloom syndrome (BS) patient and a healthy donor (WT). Tubulin is a loading control. (**B**) Western blot of extracts from U2OS cells treated with siRNA targeting firefly luciferase (siCtrl) or BLM (siBLM). H2AX is a loading control. (**C**) BLM-TopBP1 interaction does not maintain BLM stability in S phase. U2OS cells stably expressing GFP-BLM proteins were released from a thymidine block, and cycloheximide (CHX) was added for the indicated times before harvesting for western blotting. H2AX is a loading control.

Supplemental Experimental Procedures

Primary antibodies used in this study.

Target	Antibody No.	Source	Application*	Dilution
BLM	A300-110A	Bethyl Laboratories	IP, WB	1:5000
BLM-pS304	-	Dr. Yi Wang	WB	1:500
CHK1	sc8408	Santa Cruz Biotechnology	WB	1:1000
CldU	ab6326	Abcam	IF	1:400
E1A	-	Dr. Roger Grand	WB	1:15
FANCJ	4578	Cell Signaling Technology	WB	1:1000
FLAG	F1804	Sigma-Aldrich	WB	1:1000
GFP	11 814 460 001	Roche	WB	1:2000
GST	sc138	Santa Cruz Biotechnology	WB	1:2000
H2AX	ab11175	Abcam	WB	1:5000
IdU	347580	BD Biosciences	IF	1:25
Ku70	ab3114	Abcam	WB	1:400
Ku80	MS-285-P1	Thermo Scientific	WB	1:2000
MDC1	-	Dr. Grant Stewart	WB	1:1000
MLH1	ab92312	Abcam	WB	1:10,000
NBS1	ab7860	Abcam	WB	1:5000
p53	sc126	Santa Cruz Biotechnology	WB	1:1500
RMI1	ab70525	Abcam	WB	1:1000
RMI2	NBP1-89962	Novus Biologicals	WB	1:5000
TOP3A	14525-1-AP	Proteintech	WB	1:1000
TopBP1	A300-111A	Bethyl Laboratories	IP, WB	1:5000
α-Tubulin	T5168	Sigma-Aldrich	WB	1:100,000

*IF, immunofluorescence; IP, immunoprecipitation; WB, Western blotting.

Plasmids used in this study.

**Plasmid expressing**	**Vector**	**Organism**	**Details**
GFP-BLM	pEGFP-C1	Human	(Hu et al., 2001)
GFP-BLM (133-1417)	pEGFP-C1	Human	(Hu et al., 2001)
GFP-BLM (1-1222)	pEGFP-C1	Human	T1223X mutation introduced by site-directed mutagenesis into GFP-BLM
GFP-BLM (1-587)	pEGFP-C1	Human	K588X mutation introduced by site-directed mutagenesis into GFP-BLM
GFP-BLM (1-133)	pEGFP-C1	Human	T134X mutation introduced by site-directed mutagenesis into GFP-BLM
GFP-BLM (S304A)	pEGFP-C1	Human	S304A mutation introduced by site-directed mutagenesis into GFP-BLM
GFP-BLM (S338A)	pEGFP-C1	Human	S338A mutation introduced by site-directed mutagenesis into GFP-BLM
GFP-BLM	pEGFP-C1	Chicken	(Hirano et al., 2005)
GFP-BLM (S251A)	pEGFP-C1	Chicken	S251A mutation introduced by site-directed mutagenesis into GFP-BLM
GFP-TopBP1	pIRESneo2	Human	(Cescutti et al., 2010)
GFP-TopBP1 (BRCT1-K154/5A)	pIRESneo2	Human	K154/5A mutation introduced by site-directed mutagenesis into GFP-TopBP1
GFP-TopBP1 (BRCT5-K704A)	pIRESneo2	Human	K704A mutation introduced by site-directed mutagenesis into GFP-TopBP1
GFP-TopBP1 (BRCT5-K704E)	pIRESneo2	Human	K704E mutation introduced by site-directed mutagenesis into GFP-TopBP1
GFP-TopBP1 (BRCT5-W711R)	pIRESneo2	Human	W711R mutation introduced by site-directed mutagenesis into GFP-TopBP1
GFP-TopBP1 (BRCT7-K1317A)	pIRESneo2	Human	K1317A mutation introduced by site-directed mutagenesis into GFP-TopBP1
GST-TopBP1 (BRCT4+5)	pGEX-4T-1	Human	(Schmidt et al., 2008)
GST-TopBP1 (BRCT5)	Contact Wang et al.	Human	(Wang et al., 2013)
SFB-BLM	Contact Wang et al.	Human	(Wang et al., 2013)
SFB-BLM (K3A)	Contact Wang et al.	Human	(Wang et al., 2013)
SFB-BLM (S304A)	Contact Wang et al.	Human	S304A mutation introduced by site-directed mutagenesis into SFB-BLM
SFB-BLM (S338A)	Contact Wang et al.	Human	(Wang et al., 2013)

siRNAs used in this study.

Name	Target	Sequence
siCtrl	Firefly luciferase	5'-CGUACGCGGAAUACUUCGA-3'
siTopBP1-1	TopBP1	5'-ACAAAUACAUGGCUGGUUA-3'
siTopBP1-2	TopBP1	5'-CUCACCUUAUUGCAGGAGA-3'
siTopBP1-3	TopBP1	5'-GUAAAUAUCUGAAGCUGUA-3'
siTopBP1-4	TopBP1	5'-GCACAAGGUUUAAUGAGGA-3'
siBLM	BLM	5'-GCUAGGAGUCUGCGUGCGA-3'

Peptides used in this study.

Peptide	Sequence
S304	Biotin-DTDFVPPSPEEII-NH_2
pS304	Biotin-DTDFVPP[pS]PEEII-NH_2
S338	Biotin-CKEDVLSTSKDLLSKPE-NH_2
pS338	Biotin-CKEDVLST[pS]KDLLSKPE-NH_2

Peptides were found to be >95% pure after reverse phase high-performance liquid chromatography and compositions were verified by mass spectrometry.

Supplemental References

Hirano, S., Yamamoto, K., Ishiai, M., Yamazoe, M., Seki, M., Matsushita, N., Ohzeki, M., Yamashita, Y.M., Arakawa, H., Buerstedde, J.M., *et al.* (2005). Functional relationships of FANCC to homologous recombination, translesion synthesis, and BLM. The EMBO journal *24*, 418-427.

Schmidt, U., Wollmann, Y., Franke, C., Grosse, F., Saluz, H.P., and Hanel, F. (2008). Characterization of the interaction between the human DNA topoisomerase IIbeta-binding protein 1 (TopBP1) and the cell division cycle 45 (Cdc45) protein. The Biochemical journal *409*, 169-177.
